# The impact of IL28B genotype on the gene expression profile of patients with chronic hepatitis C treated with pegylated interferon alpha and ribavirin

**DOI:** 10.1186/1479-5876-10-25

**Published:** 2012-02-07

**Authors:** Zobair M Younossi, Aybike Birerdinc, Mike Estep, Maria Stepanova, Arian Afendy, Ancha Baranova

**Affiliations:** 1 Betty and Guy Beatty Center for Integrated Research, Inova Health System, Falls Church, VA, USA; 2 Center for the Study of Genomics in Liver Diseases, School of Systems Biology, College of Science, George Mason University, Fairfax, VA, USA; 3 Center for Liver Diseases and Department of Medicine, Inova Fairfax Hospital, Falls Church, VA, USA

**Keywords:** HCV, Gene Expression, Pathway Analysis, IL28B, SOCS1, IRF2, chronic hepatitis C, HCV treatment

## Abstract

**Background:**

Recent studies of CH-C patients have demonstrated a strong association between IL28B CC genotype and sustained virologic response (SVR) after PEG-IFN/RBV treatment. We aimed to assess whether IL28B alleles rs12979860 genotype influences gene expression in response to PEG-IFN/RBV in CH-C patients.

**Methods:**

Clinical data and gene expression data were available for 56 patients treated with PEG-IFN/RBV. Whole blood was used to determine IL28B genotypes. Differential expression of 153 human genes was assessed for each treatment time point (Days: 0, 1, 7, 28, 56) and was correlated with IL28B genotype (IL28B C/C or non-C/C) over the course of the PEG-IFN/RBV treatment. Genes with statistically significant changes in their expression at each time point were used as an input for pathway analysis using KEGG Pathway Painter (KPP). Pathways were ranked based on number of gene involved separately per each study cohort.

**Results:**

The most striking difference between the response patterns of patients with IL28B C/C and T* genotypes during treatment, across all pathways, is a sustained pattern of treatment-induced gene expression in patients carrying IL28B C/C. In the case of IL28B T* genotype, pre-activation of genes, the lack of sustained pattern of gene expression or a combination of both were observed. This observation could potentially provide an explanation for the lower rate of SVR observed in these patients. Additionally, when the lists of IL28B genotype-specific genes which were differentially expressed in patients without SVR were compared at their baseline, IRF2 and SOCS1 genes were down-regulated regardless of patients' IL28B genotype. Furthermore, our data suggest that CH-C patients who do not have the SOCS1 gene silenced have a better chance of achieving SVR. Our observations suggest that the action of SOCS1 is independent of IL28B genotype.

**Conclusions:**

IL28B CC genotype patients with CH-C show a sustained treatment-induced gene expression profile which is not seen in non-CC genotype patients. Silencing of SOCS1 is a negative and independent predictor of SVR. These data may provide some mechanistic explanation for higher rate of SVR in IL28B CC patients who are treated with PEG-IFN/RBV.

## Background

The most common cause of chronic liver disease, chronic hepatitis C virus (CH-C), affects approximately 170 million people worldwide [1]. Until recently, treatment of CH-C included a combination of pegylated interferon-α (PEG-IFN-α) and ribavirin (RBV). This treatment typically led to sustained virologic response (SVR) rates of 47% to 54% for previously untreated patients [2,3]. Subsequent studies have clarified that the success of this treatment is affected by several host, viral, and treatment factors. Host factors that can influence SVR include obesity, presence of cirrhosis, ethnicity, gender, and age [4-7]. Recently, a landmark genome wide association study of about 1,600 CH-C patients has identified a single nucleotide polymorphism (SNP) on chromosome 19q13, rs12979860 which was strongly associated with SVR [8]. This SNP is located upstream of the IL28B gene, encoding for IFN-λ3. Subsequently, the C/C genotype of IL28B was shown to be associated with a greater than 2 fold increase in SVR as compared with the C/T and T/T genotypes [8]. In addition, a subsequent study provided evidence for strong association of the C/C genotype with the spontaneous resolution of HCV infection [9]. Since these landmark studies, numerous lines of evidence have solidified the association between the IL28B C/C genotype and SVR in patients with CH-C. Although initially the association of IL28B C/C genotype with spontaneous clearance and SVR were noted in patients with HCV genotype 1 [10,11], more recent data have suggested a similar impact for IL28B on other HCV genotypes [12-15].

Despite the great strides in solidifying the association between IL28B genotypes and SVR, the exact underlying mechanism remains largely unknown [16-18]. In an attempt to shed some light on the potential mechanism of the allelic association of IL28B with SVR, we assessed differential gene expression in CH-C patients according to their IL28B genotypes over the course of the PEG-IFN/RBV treatment.

## Methods

The study used existing specimen repository for IL28B genotyping as well as our previously reported gene expression and associated clinical data [19]. We selected CH-C patients treated with a course of the standard doses of PEG-IFN-α2a or PEG-IFN-α2b and a weight-based dose of RBV with the duration of treatment based on HCV genotypes (N = 56). Of these 56 patients, 28 were treatment naïve and 28 were previously treated). Clinical, demographic, and laboratory data, as well as blood samples were obtained prior to treatment and during treatment. Blood samples were used for DNA and mRNA extraction as described below.

During treatment, early virologic response (EVR, as defined by > 2 log drop by week 12) and complete virologic response (complete EVR, as defined by undetectable virus by week 12) as well as post treatment sustained virologic response (SVR) rates were determined for each patient.

### Ethical approval

This study is in compliance with the Helsinki Declaration and was approved by the Inova Health Systems Institutional Review Board. Written informed consent was obtained from the participants of this study.

### DNA Extraction and Genotyping

Frozen whole blood was used for IL28B genotyping after genomic DNA extraction using QIAamp DNA Blood Mini kit (Qiagen). IL28B genotyping was performed by tetra-primer refractory mutation system PCR as described by Galmozzi et al [20]. The primers sequences were as follows: outer Fw: 5' AACTCAACGCCTCTTCCTCCT 3'; outer Rv: 5' TTCCCATACACCCGTTCCTGT 3'; inner Fw (T): 5' AGGAGCTCCCCGAAGGAGT 3'; inner Rv (G): 5' GTGCCATTCAACCCTGGTACG 3'. For each sample, we used 20-60 ng of genomic DNA for PCR (HotStarTaq Master Mix Kit; Qiagen) in test tubes containing 20 pmol of each of the four primers to amplify both the "C" and "T" alleles. Genotypes were discriminated by size via standard electrophoresis.

### RNA Extraction and Gene Expression Profiling

As previously reported [19], blood samples for each patient were collected into PAXgene™ RNA blood tubes (PreAnalytiX) prior to treatment (day 0), 1 day (day 1), 1 week (day 7), 4 weeks (day 28), and 8 weeks (day 56) after the first dosing. From the pre-treatment as well as post treatment PBMCs, total RNA was extracted, quantified and used for one step RT-PCR to profile the expression of 153 human genes that belong to various IFN-inducible and immune response related pathways. Amplification was performed with SYBR^® ^Green using 5 ng of total RNA as a template in 384-well format with a duplicate of each 15-μl reaction using Prism^® ^7900HT Sequence Detection System (Applied Biosystems). mRNA expression levels were normalized by using six housekeeping genes, a reference RNA and the ΔΔCt method [21].

### Statistical Analysis

For each time point (day 0, 1, 7, 28 and 56), differentially expressed genes were separated into up and down-regulated gene lists according to the IL28B C/C or non-C/C (T/T or T/C) genotypes. Additionally, for each time point, gene expression data were also correlated with SVR. For each cohort, means and variances of gene expression levels for each gene were calculated. Differentially expressed genes were determined using the Mann-Whitney test (p-value < 0.05) for the expression levels of each gene quantified during each of the five visits.

### Pathway Specific Analysis and Mapping

Pathways specific analyses were performed using the KEGG Pathway Painter (KPP), a novel publicly available tool (http://www.cos.gmu.edu/~gmanyam/kegg/index.html). KPP is capable of performing real-time batch painting of all relevant KEGG pathways according to differentially expressed gene lists provided by the user and placing these genes within molecular pathways previously described in Kyoto Encyclopedia of Genes and Genomes (KEGG). For each time point (day 0-day 56), the pathways encompassing SVR related genes according to IL28B SNP genotype (C/C or T*) were sorted in accordance with the pathway relevance scores, defined as the number of genes highlighted by the gene expression study and divided by the total number of genes within a given pathway. All pathways shared between two or more SVR related genes according to IL28B SNP genotype specific comparisons were automatically painted according to the direction of the gene expression change (activation or suppression). Additionally, pathways relevant to HCV pathogenesis were selected according to the previously published studies [22,23]. In order to generate a time course image of pathway involvement for SVR related genes according to IL28B SNP genotype, pathways with the highest scores were organized according to relevance scores and time course.

## Results

### Patient Population

Fifty-six patients with CH-C were included in this study. The baseline characteristics and treatment response to combination therapy are listed in Table 1.

**Table 1:** Characteristics of the patients with IL28B C/C and IL28B T* genotypes.

Characteristics	IL28B C/C	IL28B T*	All HCV patients
Number of Patients	23	33	56

Sex-Female	6 (26.1%)	15 (45.5%)	21 (37.5%)

Age	49.9 ± 4.4	47.5 ± 6.7	48.52 ± 5.96

Race, African American (#)	1 (4.3%)	8 (24.2%)	9 (16.1%)

Cirrhosis	5(22.7%)	8(25.0%)	13 (24.1%)

Obesity	13(56.5%)	16(48.5%)	29(51.8%)

Diabetes mellitus	1(4.3%)	5(15.2%)	6(10.7%)

HCV genotype 1	16 (65.2%)	27 (81.8%)	43(76.8%)

Pretreatment ALT	96 ± 59	111 ± 79	104 ± 72

Pretreatment HCV RNA (IU/mL)	5,596,354 ± 6,524,048	4,424,606 ± 5,295,969	4,893,305 ± 5,788,661

High Viral Load (> 850,000 IU/mL)	17 (77.3%)	25 (75.8%)	42 (76.4%)

No Complete EVR (p < 4.27 e^-05^)	3 (13%)	22 (68.8%)	25 (45.5%)

No EVR (p < 0.005)	1 (4.3%)	12 (37.5%)	13 (26.3%)

Sustained Virologic Response (p < 0.0022)	15 (65.2%)	8 (24.2%)	23 (41.1%)

Of the study cohort, 41% (23/56) had IL28B CC genotype and 59% (33/56) had non-CC genotype (TC/TT or T*) genotypes. In CH-C patients with IL28B C/C genotype, 65% (15/23) achieved SVR as compared to 24% (8/33) in CH-C patients with IL28B T* genotype (p < 0.05).

Restricting the analysis only to HCV genotype 1 patients (43/65), the association of IL28B genotypes with SVR remained unchanged (SVR: IL28B C/C: 62.5%, IL28B T*: 22.2%, p < 0.05).

IL28B T* genotype was more prevalent in African Americans with CH-C (p < 0.05) as compared to all other ethnic groups. Cirrhosis and female gender were negatively associated with SVR only in the IL28B C/C cohort (p < 0.0085 and p < 0.0037, respectively).

### Gene Expression Profiling

A total of 150 human genes analyzed in individual qRT-PCR assays were profiled across five time points (Day 0; Day1; Day7; Day28; Day56) along the PEG-IFN/RVB treatment. The complete list of analyzed genes could be found in the Additional File 1. Gene expression profiles were used to compare IL28B C/C and IL28B T*cohorts across all time points for the duration of treatment. For each of the time points, the genes were separated into up and down regulated groups according to the genotype. Additionally, for each time point, genotype-specific groups of up- and down-regulated genes which were associated with SVR were identified (Table 2). After the parsing of the data, gene lists were entered into KPP for pathway analysis. These analyses were repeated for CH-C patients with HCV genotype 1 only.

**Table 2:** Genes differentially up- or downregulated in SVR in IL28B CC and IL28T* cohorts over the course of the treatment period (P < 0.05).

Alelle	Day 0		Day 1		Day 7		Day 28		Day 56	
	**Up**	**Down**	**Up**	**Down**	**Up**	**Down**	**Up**	**Down**	**Up**	**Down**

**IL28B CC**		None					ATP6V0B			BST2
	IL1B IRF2 NFKB2 PRDM1 SOCS1		CCL4 IFITM1 SSBP1	LIPA PRKRA	ADAR ATP6V0B BTG1 CCL3 CCL4 CREB1 ICAM1 IKBKG IRF1 LYN PLAUR PRKRIR RNASEL SOCS1 STAT3 STAT6 TAP2	FYN IRF3	EIF2AK2 FAS GBP1 GTPBP2 IFI35 IFI44 IFI44L IFIT1 IFIT2 IFIT5 IFITM3 IRF7 SELL SP110 TAP2 TRIM34	DHX9 FYN IRF3 LIPA	IFITM3	CD81 FYN IKBKB IRF3 MX1 PSME2 TRIM14 TRIM26 TRIM34

IL28B T*	AIM2 IKBKE IRF2 LCK SOCS1	STAT6	IRF2 LCK TRAF6	NMI YARS	BTG1 CREB1 FAS GMPR HIF1A IFNAR1 IRF2 SELL SOCS1 TAP1 TGFB1 TRADD TRAF6	IL10 SHFM1 YARS	IFNAR1	NMI PRKRA	CREB1	CTLA4 IFI30 IRF5

### Analysis of the Pathways Associated with SVR across Both IL28B genotypes

The KPP analysis of genes associated with SVR in both IL28B CC and IL28T* cohorts highlighted several pathways that may play an important role in determining patients' response to interferon-based treatment. These pathways were independently identified for all HCV genotypes as well as specifically for HCV genotype 1 sub-cohort.

When IL28B C/C and IL28B T* genotypes were compared to each other, the variation between genes grouped in "Hepatitis C infection" pathway was the most striking pathway. This pathway was not only pre-activated in the IL28B T* patients, but it was also associated with a distinct pattern of activation and suppression as compared to the IL28B C/C genotype (Table 3).

**Table 3:** Hepatitis C pathway related genes are differentially regulated in IL28B CC and IL28 T* genotypes over the course of the treatment.

Genotype	Day 0	Day 1	Day 7	Day 28	Day 56
**IL28B CC**	**(0)**	**(0)**	**(5)** RNASEL **IRF3** IKBKG IRF1 STAT3	**(3)** IRF3 EIF2AK2 (PKR) IFIT1	**(3)** **CD81** **IRF3** **IKBKB**

IL28B T*	**(1)** IKBKE	**(1)** TRAF6	**(3)** TRAF6 IFNAR1 TRADD	**(1)** IFNAR1	**(0)**

Upregulated genes are shown in bold; Downregulated genes are shown in regular font.

**Figure 1:**
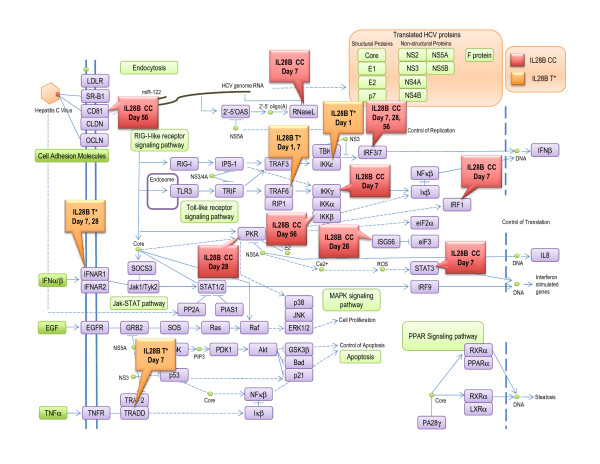
**A map of HCV pathway**. Genes differentially activated during PEG-IFN/RBV treatment in patients with IL28B T* genotype are highlighted in green and with IL28B CC genotype are highlighted in blue.

When the lists of genes highlighted in IL28B C/C vs. T* comparisons were cross-referenced with the list of SVR-related genes that has been previously reported [20], the sustained pattern of treatment-induced gene expression changes during the entire treatment period was observed only in the IL28B CC patients. On the other hand, in the T* cohorts, the pre-activation of SVR associated genes was observed. More specifically, gene expression changes in the Hepatitis C Pathway (Figure 1) were detected in patients with IL28B C/C genotype after a week of treatment. Genes associated with this pathway continued to be differentially expressed at 28 days and 56 days after treatment was initiated. Although, in CH-C patients with IL28B T*, genes associated with this pathway were already activated prior to treatment (Day 0) as well as early (24 hours) after treatment, this pathway became less pronounced after treatment (Figure 1, Table 3).

**Figure 2:**
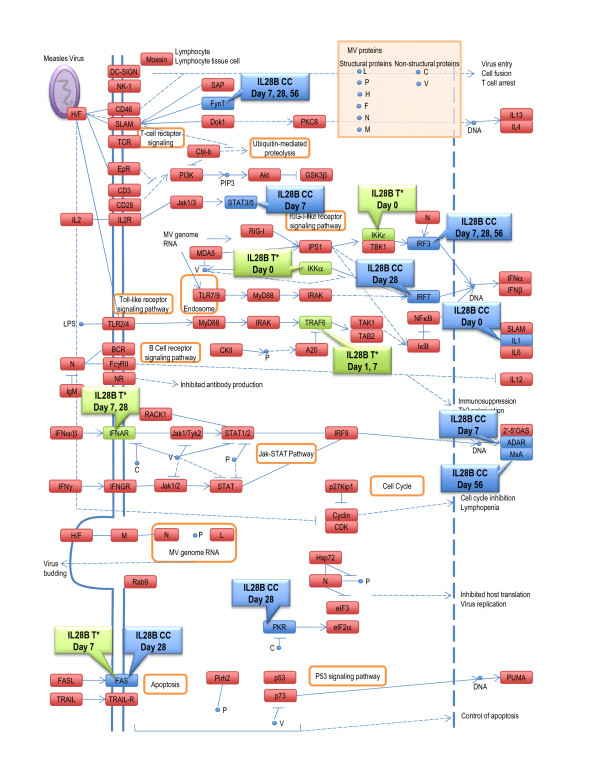
**A map of measles pathway**. Genes differentially activated during PEG-IFN/RBV treatment in patients with IL28B T* genotype are highlighted in green and with IL28B CC genotype are highlighted in blue.

Additionally, we identified two other pathways that could potentially differentiate IL28B C/C from T* genotypes in terms of antiviral response. These pathways were the Measles Pathway (Figure 2) and Chemokine Signaling Pathway (Table 4).

**Table 4:** Genes involved in the Cytokine Signaling Pathway related genes are differentially regulated in IL28B CC and IL28 T* genotypes over the course of the treatment.

Genotype	Day 0	Day 1	Day 7	Day 28	Day 56
**IL28B CC**	**(1)** IL1B	**(0)**	**(4)** ADAR FYN IRF3 STAT3	**(5)** EIF2AK2 **FAS** **FYN** **IRF3** IRF7	**(3)** **FYN** **IRF3** **MX1**

IL28B T*	**(1)** IKBKE	**(1)** TRAF6	**(3)** FAS IFNAR1 TRAF6	**(1)** IFNAR1	**(0)**

Upregulated genes are shown in bold; Downregulated genes are shown in regular font.

### Analysis of the Pathways Associated with Treatment-failure Across Both IL28B Genotypes

**Figure 3:**
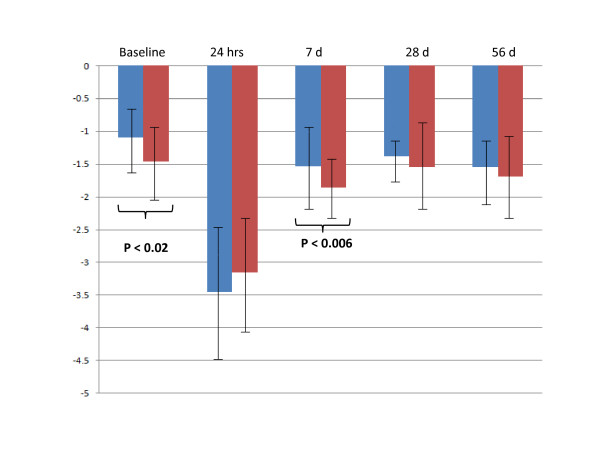
**The time course of the changes in SOCS1 expression levels in patients who subsequently achieved or not achieved SVR**. Blue columns: patients who achieved SVR (N = 23). Red columns: patients who not achieved SVR (N = 33).

**Figure 4:**
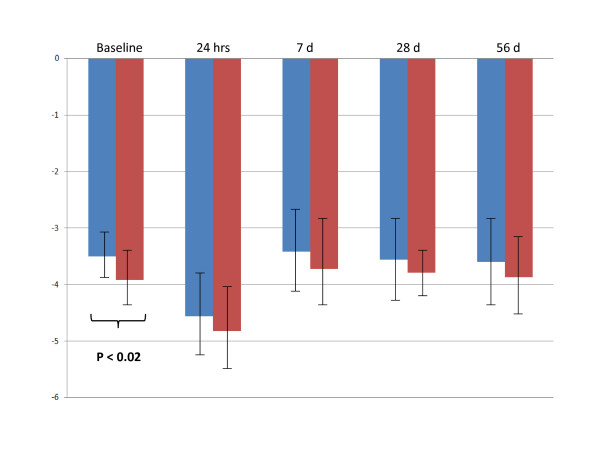
**The time course of the changes in IRF2 expression levels in patients who subsequently achieved or not achieved SVR**. Blue columns: patients who achieved SVR (N = 23). Red columns: patients who not achieved SVR (N = 33).

The lists of genes differentially expressed in CH-C patients who did not achieve SVR despite having IL28B C/C genotype (8/23) was compared to IL28B T* genotype patients (25/33) who also did not achieve SVR (Table 2). Remarkably, prior to initiation of treatment, IRF2 and SOCS1 genes were independently down-regulated in both groups of IL28B patients. Although these CH-C patients had different IL28 B genotypes, they all shared the common characteristic of not achieving SVR and having pre-treatment down-regulation of the IRF2 and SOCS1 genes. Neither SOCS1, nor IRF2 expression levels were different when IL28B C/C genotype and IL28B T* genotype cohorts were compared. The time course of the changes in SOCS1 and IRF2 expression levels in patients who subsequently achieved or not achieved SVR is shown at Figures 3 and 4, respectively.

## Discussion

Although the recent approval of Direct Antiviral Agents (DAA) for treatment of CH-C will change the treatment course of CH-C patients, over the past decade, the combination of PEG-INF/RBV has been the standard treatment for CH-C with 47% to 54% SVR rates [24]. Recently, the discovery of IL28B, as a strong predictor of SVR, has brought a great deal of enthusiasm. Multiple studies have shown that CH-C patients carrying the C/C genotype have a two-fold greater chance of SVR than patients with the T* genotype (8, 10, 11). Our results are consistent with these data, showing that SVR rates are 2-3 times higher in CH-C patients with IL28B CC genotype.

Additional research has shown that IL28B T* genotype is more common in African American patients with CH-C [8,9] who typically don't respond favorably to PEG-IFN/RBV [25]. Consistent with these data, our study showed a higher prevalence of the T* allele in African American patients with CH-C (Table 1). These data confirm the role of IL28B genotypes in SVR and provide evidence for validation of our data [26,27].

Despite the strong association between IL28B CC genotype and SVR, the underlying mechanism of IL28 B in determining SVR remains unknown. It is postulated that interferon lambda may produce a better immunologic profile that could favor viral clearance [28]. Recent study by Honda and co-authors demonstrated that the expression of hepatic interferon-stimulated genes is strongly associated with both treatment response and genetic variation of IL28B [29]. In our study, we assessed the impact of PEG-IFN/RBV on the gene expression profiles in PBMCs of patients with CH-C during treatment and analyzed these data according to their IL28B genotype status as well as their SVR outcome. We used previously collected gene expression data from a cohort of CH-C patients who were undergoing treatment with PEG-IFN/RBV [19] and analyzed the gene expression profile of these patients according to these patients' IL28B genotype status. Furthermore, this gene expression analysis was coupled with pathway analyses offering a novel approach to the interpretation of gene expression data [26]. This approach provided us with further advancement over previously published time course studies of IFN-inducible genes in PBMCs of HCV patients treated with PEG-IFN/RBV [30,31].

Our approach was validated by the fact that the most commonly activated pathway in our analysis was the Hepatitis C Pathway itself [Figure 1] Analysis of this pathway revealed two interesting observations. First, CH-C patients with IL28B T* genotype seem to have baseline activation of the Hepatitis C Pathway, via the increased expression of the IKBKE gene known to be essential for regulating antiviral signaling, whereas the CH-C patients with the IL28B C/C genotype showed activation in this pathway only after the initiation of treatment (Day 7). Second, we observed that IL28B C/C genotype patients with CH-C maintained a sustained involvement of this pathway throughout the entire 56 days of treatment with PEG-IFN/RBV; The IL28B T* patients, on the other hand, showed activation at baseline, with minimal involvement past day 7 after the initiation of treatment (Table 3).

Our study also highlighted the Chemokine Signaling as a pathway of interest. In fact, patients with CH-C IL28B CC genotype showed a clear treatment induced involvement of this pathway, whereas IL28B T* genotype failed to show any treatment induced activation of this pathway (Table 4).

It should be noted that IL28B polymorphisms has not been as strongly associated with treatment outcomes for patients with chronic hepatitis B or human immunodeficiency virus to date [32,33]. However, it is possible that HCV shares its regulatory pathways with other types of viruses. In this context, our observation that the pathway related to measles infection is especially intriguing [Figure 2]. In fact, it has been recently shown that the V protein of the measles virus is a potent inhibitor of IFN-lambda which is encoded by the IL28B gene [34]. It may be interesting to assess any potential association of IL28B and SVR in CH-C patients according to their previous exposure to measles virus or MMR vaccine.

To gain additional insights into the mechanisms of genotype-specific treatment-failure, we compared the lists of genes which were differentially expressed in patients who could not achieve SVR, irrespective of their Il28B genotype status. Remarkably, when gene expression patterns were compared at baseline, IRF2 and SOCS1 genes were independently identified as being down-regulated in all CH-C patients who failed treatment, regardless of their IL28B genotypes. Interestingly, transcriptional silencing of the SOCS1 gene in the liver has been suggested as a potential reason for treatment failure in CH-C patients as well as in the mouse model of chronic HCV infection [35]. In both instances, silencing of SOCS1 led to permanent activation of the JAK-STAT signaling pathway [35]. Additionally, hepatocytes lacking SOCS-1 exhibit a prolonged response to IFNγ [36]. Conversely, when SOCS1 was over-expressed, the propagation of the signal through hepatic interferon-dependent pathways is abrogated [36,37]. In fact, this interferon pathway shutdown also influences IFN-λ [37]. Our data collected using PBMCs indicate that the action of SOCS1 is independent of IL28B genotype. Furthermore, our data suggest that CH-C patients who do not have the SOCS1 gene silenced have a better chance of achieving SVR. This hypothesis is supported by our previous observation that the lack of SOCS1 suppression in PBMCs could serve as a predictor of SVR, independent of the viral genotype or treatment status [38].

This study has two major limitations, an enrollment of patients infected by different HCV genotypes and of different ethnicity and evaluation of peripheral blood samples instead of hepatic tissue. However, it is important to note that we carried out our gene expression pathway analyses with all HCV genotypes and then analyzed the data separately for HCV genotype 1 patients. This was done to assess the impact of HCV genotype- specific response according to IL28B alleles. Nevertheless, the results of our pathway analyses for both HCV genotype groups were very similar, confirming that IL28B acts independent of HCV genotype.

## Conclusions

In conclusion, the most striking difference between the response patterns of IL28B C/C and T* genotypes to antiviral treatment across all pathways, was the sustained pattern of treatment-induced gene expression in IL28B C/C patients. In the case of IL28 T* genotype, the pre-activation of this pathway, the lack of sustained activation post treatment or a combination of both, could provide an explanation for their high rate of treatment failure. Further studies are needed to clarify the mechanism of IL28B genotyping and its impact on treatment response.

## Competing interests

The authors declare that they have no competing interests.

## Authors' contributions

ABar and ZY designed the study and edited the manuscript. AA collected the samples. ME performed genotyping. MS performed statistical analysis. ABar generated SOCS1 graphs. ABir performed KPP analysis and drafted a manuscript. All authors read and approved the final manuscript.

## Authors' information

ABar is an Associate Professor at the School of Systems Biology, College of Science, George Mason University (SSB COS GMU). ABir is Research Assistant Professor at SSB COS GMU. ME and AA are Research Associates at Betty and Guy Beatty Center for Integrated Research, Inova Health System. ZY is a Chairman, Department of Medicine, Inova Fairfax Hospital and Vice President for Research, Inova Health System

## Supplementary Material

Additional file 1 **The complete list of analyzed genes**. This file lists all genes analyzed using qRT-PCR and includes gene names, gene IDs and the biological functions of these genes. Click here for file
