# Supplementary material for: The impact of IL28B genotype on the gene expression profile of patients with chronic hepatitis C treated with pegylated interferon alpha and ribavirin
Source: J Transl Med. 2012 Feb 7;10:25. doi: 10.1186/1479-5876-10-25 (PMC3296607; doi:10.1186/1479-5876-10-25)
Supplement: Additional file 1 — The complete list of analyzed genes. This file lists all genes analyzed using qRT-PCR and includes gene names, gene IDs and the biological functions of these genes. [file 1479-5876-10-25-S1.DOC]

| Gene Name | Gene ID | Biological Function |
| --- | --- | --- |
| **JAK-STAT SIGNALING PATHWAY** | | |
| Janus kinase 1 | JAK1 | Is involved in the interferon-α/β and -γ signal transduction pathways. The reciprocal interdependence between JAK1 and TYK2 activities in the interferon- α pathway, and between JAK1 and JAK2 in the interferon- γ pathway. Couples cytokine ligand binding to tyrosine phosphorylation of various known signaling proteins and of STAT family of transcription factors. |
| Janus kinase 2 | JAK2 | A protein tyrosine kinase involved in a specific subset of cytokine receptor signaling pathways. It has been found to be Is required for responses to gamma interferon. |
| Signal transducer and activator of transcription 1, 91kDa | STAT1 | In response to cytokines and growth factors, STAT family members are phosphorylated by the receptor associated kinases, and then form homo- or heterodimers that translocate to the cell nucleus where they act as transcription activators. This protein can be activated by various ligands including interferon-alpha, interferon-gamma, EGF, PDGF and IL6. |
| Signal transducer and activator of transcription 2 | STAT2 | Forms a complex with STAT1 and IRF9. Interacts with EP300 and plays role in IFN-α response. |
| Signal transducer and activator of transcription 3 | STAT3 | Activated in response to IFNs, EGF, IL5, IL6, HGF, LIF and BMP2. Plays a key role in many cellular processes such as cell growth and apoptosis. |
| Signal transducer and activator of transcription 4 | STAT4 | Mediates responses to IL12 in lymphocytes. Regulates the differentiation of T helper cells. |
| Signal transducer and activator of transcription 5A | STAT5A | Mediates the responses of IL2, IL3, IL7, GM-CSF, erythropoietin, thrombopoietin, and growth hormones. Induce the expression of anti-apoptotic BCL2L1/BCL-X(L). |
| Signal transducer and activator of transcription 5B | STAT5B | Mediates the signal transduction triggered by various cell ligands, such as IL2, IL4, CSF1, and different growth hormones. It has been shown to be involved in diverse biological processes, such as TCR signaling, apoptosis, adult mammary gland development, and sexual dimorphism of liver gene expression. |
| Signal transducer and activator of transcription 6 | STAT6 | Exerts IL4 mediated biological responses. Induce the expression of anti-apoptotic BCL2L1/BCL-X(L). Plays role in differentiation of Th2 cells, expression of cell surface markers, and class switch of immunoglobulins. |
| Protein inhibitor of activated STAT | PIAS1 | It inhibits STAT1-mediated gene activation and the DNA binding activity, binds to Gu protein/RNA helicase II/DEAD box polypeptide 21, and interacts with androgen receptor (AR). It functions in testis as a nuclear receptor transcriptional coregulator and may have a role in AR initiation. |
| N-myc (and STAT) interactor | NMI | Interacts with NMYC, CMYC and all STATs except STAT2. Augments STAT-mediated transcription in response to cytokines IL2 and IFN-gamma. |
| Cytotoxic T-lymphocyte-associated protein 4/CD152 | CTLA4 | Negative regulator of T-cell proliferation. Interacts with STAT5 and inhibits STAT5-mediated transcription. |
| **TRANSCRIPTION FACTORS (NON-STAT) and CO-ACTIVATORS** | | |
| E1A binding protein p300 | EP300 | Stimulates transcription through activation of CREB. Co-activator of HIF1A, IRF3, STAT2 and STAT3. Stimulates Foxo1-induced transcription of IGF-binding protein-1. Essential for COX-2 transcriptional activation by proinflammatory mediators. Repressed by SIRT1. Regulates fibronectin expression via PARP and NF-kappaB activation in diabetes. |
| HIF1A hypoxia-inducible factor 1, α subunit | HIF1A | Plays an essential role in cellular and systemic homeostatic responses to hypoxia. |
| cAMP responsive element binding protein 1 | CREB1 | Induces transcription of genes in response to hormonal stimulation of the cAMP pathway. Activated by HCV through Ca2+/calmodulin-dependent protein kinase. |
| Class II, major histocompatibility complex, transactivator | CIITA | Acts as a positive regulator of class II major histocompatibility complex gene transcription, and is referred to as the "master control factor" for the expression of these genes. |
| Promyelocytic leukemia (PML) protein | PML | This phosphoprotein localizes to nuclear bodies where it functions as a transcription factor and tumor suppressor. Its expression is cell-cycle related and it regulates the p53 response to oncogenic signals. |
| Nuclear factor, interleukin 3 regulated (E4BP4) | NFIL3 | Binds and represses viral promoter sequences. Involved in the commitment to cell survival versus apoptosis early B-cell progenitors and the anti-inflammatory response. A "delayed-early" IL-3-responsive gene. |
| SP110 nuclear body protein | SP110 | This gene is a member of the SP100/SP140 family of nuclear body proteins and encodes a leukocyte-specific nuclear body component. The protein can function as an activator of gene transcription and may serve as a nuclear hormone receptor coactivator. In addition, it has been suggested that the protein may play a role in ribosome biogenesis and in the induction of myeloid cell differentiation. |
| CCAAT/enhancer binding protein (C/EBP), beta | CEBPB | Is important in the regulation of genes involved in immune and inflammatory responses and has been shown to bind to the IL-1 response element in the IL-6 gene, as well as to regulatory regions of several acute-phase and cytokine genes. |
| **OTHER KINASES, PHOSPHATASES AND OTHER SIGNAL TRANSDUCERS (NON-JAK)** | | |
| Lymphocyte-specific protein tyrosine kinase | LCK | Encodes a key signaling molecule in the selection and maturation of developing T-cells. LCK localizes to the plasma membrane and pericentrosomal vesicles, and binds to cell surface receptors, including CD4 and CD8, and other signaling molecules. |
| LYN kinase (JTK8) | LYN | Negatively regulates signaling pathways through phosphorylation of inhibitory receptors, enzymes, and adaptors. Somewhat paradoxically, it is also a key mediator in several pathways of B cell activation, such as CD19 and CD180. Consequences of Lyn activity are context dependent. |
| FYN kinase | FYN | Has been implicated in the control of cell growth. The protein associates with the p85 subunit of phosphatidylinositol 3-kinase and interacts with the fyn-binding protein. |
| Ras homolog gene family, member C | RHOC | Rho proteins promote reorganization of the actin cytoskeleton and regulate cell shape, attachment, and motility. The protein encoded by this gene is prenylated at its C-terminus, and localizes to the cytoplasm and plasma membrane. It is thought to be important in cell locomotion. |
| SMAD family member 3 | SMAD3 | SMAD proteins are signal transducers and transcriptional modulators that mediate multiple signaling pathways. This protein functions as a transcriptional modulator activated by transforming growth factor-beta. |
| Phosphatase and tensin homolog | PTEN | A phosphatidylinositol-3,4,5-trisphosphate 3-phosphatase that negatively regulates intracellular levels of phosphatidylinositol-3,4,5-trisphosphate in cells and negatively regulates AKT/PKB signaling pathway. |
| **CYTOKINES, CHEMOKINES AND THEIR RECEPTORS** | | |
| chemokine (C-C motif) ligand 2 (MCP-1) | CCL2 | Displays chemotactic activity for monocytes and basophils but not for neutrophils or eosinophils. It has been implicated in the pathogenesis of diseases characterized by monocytic infiltrates, like psoriasis, rheumatoid arthritis and atherosclerosis. It binds to chemokine receptors CCR2 and CCR4. |
| chemokine (C-C motif) ligand 5 (RANTES) | CCL5 | Functions as a chemoattractant for blood monocytes, memory T helper cells and eosinophils. It causes the release of histamine from basophils and activates eosinophils. This cytokine is one of the major HIV-suppressive factors produced by CD8+ cells. It functions as one of the natural ligands for the chemokine receptor CCR5. |
| Chemokine (C-C motif) ligand 3 (MIP-1α) | CCL3 | Involved in the acute inflammatory state through the recruitment and activation of polymorphonuclear leukocytes |
| Chemokine (C-C motif) ligand 4 (MIP-1β) | CCL4 | Propagates inflammation. Recruits NK cells from circulating peripheral blood. |
| Chemokine (C-X-C motif) ligand 10 | CXCL10 | Binding of this protein to CXCR3 results in pleiotropic effects, including stimulation of monocytes, natural killer and T-cell migration, and modulation of adhesion molecule expression |
| Chemokine (C-C motif) receptor 1 | CCR1 | The ligands of this receptor include macrophage inflammatory protein 1 alpha (MIP-1 alpha), regulated on activation normal T expressed and secreted protein (RANTES), monocyte chemoattractant protein 3 (MCP-3), and myeloid progenitor inhibitory factor-1 (MPIF-1). |
| Interleukin 10 | IL10 | Down-regulates the expression of Th1 cytokines, MHC class II antigens, and costimulatory molecules on macrophages. Enhances B cell survival, proliferation, and antibody production. Blocks NF-kappa B activity. Regulates JAK-STAT signaling pathway. Blockade of IL-10R converts a chronic viral infection into an acute one and prevents the functional exhaustion of memory T cells. The higher levels of IL-10 are associated with a higher risk of an inefficient clearance of the HCV. |
| Interleukin 1β | IL1B | Important mediator of the inflammatory response. Involved in a cell proliferation, differentiation, and apoptosis. Induces cyclooxygenase-2 (PTGS2/COX2). |
| Interleukin 15 | IL15 | Regulates T and natural killer cell activation and proliferation. Shares many biological activities with IL2. The number of CD8+ memory cells is shown to be controlled by a balance between this cytokine and IL2. Induces the activation of JAK kinases, STAT3, STAT5, and STAT6. Induce the expression of anti-apoptotic BCL2L1/BCL-X(L). |
| Interleukin 15 receptor α | IL15RA | Receptor that specifically binds IL15 with high affinity. |
| Interleukin 8 | IL8 | One of the major mediators of the inflammatory response. This chemokine is secreted by several cell types. It functions as a chemoattractant, and is also a potent angiogenic factor. |
| Interleukin 18 (interferon-gamma-inducing factor) | IL18 | A proinflammatory cytokine that augments natural killer cell activity in spleen cells, and stimulates interferon gamma production in T-helper type I cells. |
| Platelet factor 4 (CXCL4) | PF4 | A strong chemoattractant for neutrophils and fibroblasts |
|  |  |  |
| **GROWTH FACTORS AND THEIR RECEPTORS** | | |
| Platelet-derived growth factor α | PDGFA | Mitogenic factor for cells of mesenchymal origin. The decrease in PDGF is shown to be associated with HCV elimination. |
| Platelet-derived growth factor β | PDGFB | This gene product can exist either as a homodimer (PDGF-BB) or as a heterodimer with the platelet-derived growth factor alpha polypeptide (PDGF-AB), where the dimers are connected by disulfide bonds. |
| Transforming growth factor β 1 | TGFB1 | Negative autocrine growth factor. Dysregulation may result in apoptosis. Suppresses the proliferation and cytotoxicity of natural killer (NK) cells which play critical roles in resolving HCV infection. |
| **TOLL-LIKE RECEPTORS** | | |
| Toll-like receptor 2 | TLR2 | This gene is expressed most abundantly in peripheral blood leukocytes, and mediates host response to Gram-positive bacteria and yeast via stimulation of NF-kappaB. |
| Toll-like receptor 4 | TLR4 | This receptor is most abundantly expressed in placenta, and in myelomonocytic subpopulation of the leukocytes. It has been implicated in signal transduction events induced by lipopolysaccharide (LPS) found in most gram-negative bacteria. Mutations in this gene have been associated with differences in LPS responsiveness. |
| Toll-like receptor 7 | TLR7 | Key mediator of innate immunity. Agonists of TLR7 have been shown to have clinical efficacy in HCV patients. |
| **SUPPRESSORS OF CYTOKINE SIGNALING** | | |
| Suppressor of cytokine signaling 1 | SOCS1 | Cytokine-induced STAT inhibitor. Functional cross-modulation between SOCS proteins can stimulate cytokine signaling. Negative regulator in TNF-induced inflammation. Associated with and inhibits the insulin receptor. Inhibited by Hepatitis C virus core protein. Inhibits expression of the antiviral proteins 2',5'-OAS and MxA. |
| Suppressor of cytokine signaling 6 | SOCS6 | Cytokine-induced STAT inhibitor. Functional cross-modulation between SOCS proteins can stimulate cytokine signaling. Induced by insulin. Associated with and inhibits the insulin receptor. Decreases the levels of the STAT3 protein in the nucleus. |
| **DIRECT EFFECTORS AND REGULATORS OF INTERFERON SIGNALING** | | |
| Bone marrow stromal cell antigen 2/ tetherin | BST2 | Interferon-induced BST2/Tetherin is a broadly acting antivitral protein. It tethers mature viral particles to the plasma membrane. BST2 also inhibits release of enveloped viruses. May play a role in pre-B-cell growth |
| PR domain containing 1, with ZNF domain | PRDM1 | This gene encodes a protein that acts as a repressor of beta-interferon gene expression. The protein binds specifically to the PRDI (positive regulatory domain I element) of the beta-IFN gene promoter. Transcription of this gene increases upon virus induction. |
| C1orf38 chromosome 1 open reading frame 38 | C1ORF38 | Interferon-induced involved in differentiation processes |
| Pprotein kinase, interferon-inducible double stranded RNA dependent activator | PRKRA | This gene encodes a protein kinase activated by double-stranded RNA which mediates the effects of interferon in response to viral infection. |
| Protein-kinase, interferon-inducible double stranded RNA dependent inhibitor | PRKRIR | Cellular inhibitor of the RNA-dependent protein kinase (PKR). Regulates the molecular co-chaperone P58IPK. |
| Eukaryotic translation initiation factor 2-alpha kinase 2 (PKR) | EIF2AK2 | Plays a stimulus- and virus-dependent role in apoptotic death and virus multiplication in human cells. |
| Adenosine deaminase, RNA-specific (IFI4) | ADAR | Enzyme responsible for RNA editing by site-specific deamination of adenosines. |
| Interferon-α inducible protein 6 | IFI6 | Directly suppresses HCV replication. |
| Interferon-γ inducible protein 16 | IFI16 | Interacts with p53 and retinoblastoma-1. Modulates p53 function. Inhibits cell growth in the Ras/Raf signaling pathway. |
| Interferon-induced protein 44 | IFI44 | The interferon-alpha (IFN-alpha)-inducible protein associated with HCV infection |
| Interferon-induced protein 44-like | IFI44L | Unclear |
| Absent in melanoma 2 | AIM2 | Is a member of the IFI20X /IFI16 family. May control cell proliferation. Is induced by Interferon-gamma |
| Interferon-α inducible protein 27 | IFI27 | Expression in liver predicts treatment response in HCV patients. Protein is localized to the nuclear envelope |
| Interferon-γ inducible protein 30 | IFI30 | Lysosomal thiol reductase that at low pH can reduce protein disulfide bonds. Has an important role in MHC class II-restricted antigen processing. |
| Interferon-induced protein 35 | IFI35 | Involved in cytokine signaling. Stabilized by NMI protein and destabilized by CKIP-1. Forms complexes with transcription factor B-ATF. |
| Myxovirus (influenza virus) resistance 1 (IFI78, MxA)) | MX1 | Dynamin-like large GTPases involved in intracellular vesicle trafficking and organelle homeostasis. Detect viral infection by sensing nucleocapsid-like structures. As a consequence, these viral components are trapped and sorted to locations where they become unavailable for the generation of new virus particles. |
| Myxovirus (influenza virus) resistance 2 (MxB) | MX2 |
| Interferon regulatory factor 1 | IRF1 | Serves as an activator of interferons alpha and beta transcription, and in mouse it has been shown to be required for double-stranded RNA induction of these genes. IRF1 also functions as a transcription activator of genes induced by interferons alpha, beta, and gamma. Further, IRF1 has been shown to play roles in regulating apoptosis and tumor-suppression. |
| Interferon regulatory factor 2 | IRF2 | Competitively inhibits the IRF1-mediated transcriptional activation of interferons α and β, and presumably other genes that employ IRF1 for transcription activation. Also functions as a transcriptional activator of histone H4. |
| Interferon regulatory factor 3 | IRF3 | IRF3 is found in an inactive cytoplasmic form that upon serine/threonine phosphorylation forms a complex with CREBBP. This complex translocates to the nucleus and activates the transcription of interferons α and β, as well as other interferon-induced genes. |
| Interferon regulatory factor 4 | IRF4 | One of the lymphoid-specific components that control the ability of T lymphocytes to produce a distinctive array of cytokines. Modulates the efficiency of the Fas-mediated death signal. |
| Interferon regulatory factor 5 | IRF5 | IRF5 plays a critical role in IFN- α and β production induced by conventional TLR7 and TLR9 ligands. Activated by expression of TANK-binding kinase 1 and by inhibitor of NF-kappaB kinase-epsilon. |
| Interferon regulatory factor 7 | IRF7 | Plays a role in the transcriptional activation of virus-inducible cellular genes, including interferon beta chain genes. Inducible expression of IRF7 is largely restricted to lymphoid tissue. |
| Interferon regulatory factor 8 | IRF8 | Transcription factor that regulate expression of genes stimulated by IFN α and β in viral infection. Mice with IRF8 deletion have defects in the macrophage function, including the ability to induce interleukin-12 (IL-12) p40 and some IFN-gamma-responsible genes. |
| ISG15 ubiquitin-like modifier | ISG15 | Ubiquitin homolog that is rapidly up-regulated after viral infection, and it conjugates to a wide array of host proteins. Previously shown to be differentially expressed between HepC treatment responders and all nonresponders. |
| Interferon stimulated exonuclease gene 20kDa | ISG20 | An interferon-inducible 3'-5' exonuclease that inhibits replication of several human and animal RNA viruses. |
| Interferon (alpha, beta and omega) receptor 1 | IFNAR1 | Binding and activation of the receptor stimulates Janus protein kinases, which in turn phosphorylate several proteins, including STAT1 and STAT2. The encoded protein also functions as an antiviral factor. |
| Interferon (alpha, beta and omega) receptor 2 | IFNAR2 | A type I membrane protein that forms one of the two chains of a receptor for interferons alpha and beta. Binding and activation of the receptor stimulates Janus protein kinases, which in turn phosphorylate several proteins, including STAT1 and STAT2. |
| Interferon gamma receptor 2 | IFNGR2 | Non-ligand-binding beta chain of the gamma interferon receptor. |
| Interferon gamma receptor 1 | IFNGR1 | Ligand-binding chain of the heterodimeric gamma interferon receptor. |
| Interferon-induced protein with tetratricopeptide repeats 1 | IFIT1 | [An antiviral protein that recognizes 5'-triphosphate RNA.](http://www.ncbi.nlm.nih.gov/pubmed/21642987/) |
| Interferon-induced protein with tetratricopeptide repeats 2/IFI54 | IFIT2 | Previously shown to be upregulated in PBMCs after 120 h of continuous PEG-IFN-α 2b treatment. Is induced by either IRF-3 or IFN stimulated gene factor 3 (ISGF3). |
| Interferon-induced protein with tetratricopeptide repeats 3 | IFIT3 | [Potentiates antiviral signaling by bridging MAVS and TBK1.](http://www.ncbi.nlm.nih.gov/pubmed/21813773/) |
| Interferon-induced protein with tetratricopeptide repeats 5 | IFIT5 | Not clear |
| Interferon induced transmembrane protein 1 | IFITM1 | Restricts HIV, filoviruses, SARS coronavirus, and influenza A |
| Interferon induced transmembrane protein 2 (1-8D) | IFITM2 | Not clear. Inhibits viral infections. |
| Interferon induced transmembrane protein 3 | IFITM3 | Not clear. Inhibits viral infections. |
| Allograft inflammatory factor 1 | AIF1 | Is induced by cytokines and interferon. Is involved in negative regulation of growth of vascular smooth muscle cells, which contributes to the anti-inflammatory response to vessel wall trauma. Mediates activation of macrophages. |
| 2',5'-oligoadenylate synthetase 1, 40/46kDa (IFI4) | OAS1 | Is induced by interferons and uses adenosine triphosphate in 2'-specific nucleotidyl transfer reactions to synthesize 2',5'-oligoadenylates (2-5As) that activates latent RNase L, which results in viral RNA degradation and the inhibition of viral replication. |
| 2'-5'-oligoadenylate synthetase 2, 69/71kDa | OAS2 |
| 2'-5'-oligoadenylate synthetase 3, 100kDa | OAS3 |
| 2'-5'-oligoadenylate synthetase-like | OASL | Not clear |
| Ribonuclease L | RNASEL | A component of the interferon-regulated 2-5A system that functions in the antiviral and antiproliferative roles of interferons. |
| Guanylate binding protein 1, interferon-inducible, 67kDa | GBP1 | Binds guanine nucleotides (GMP, GDP, and GTP). Is selectively induced by inflammatory cytokines and is an activation marker of endothelial cells during inflammatory diseases. |
| Guanylate binding protein 2, interferon-inducible | GBP2 | Binds guanine nucleotides (GMP, GDP, and GTP). |
| Phospholipid scramblase 1 | PLSCR1 | an IFN-stimulated gene. Provides a mechanism for amplifying and enhancing the IFN response through increased expression of a select subset of potent antiviral genes |
| **PROTEIN DEGRADATION AND MHC PROCESSING** | | |
| Proteasome subunit, beta type, 8 (large multifunctional peptidase 7) | PSMB8 | A member of the proteasome B-type family. Expression of PSMB8 is induced by gamma interferon. Replaces catalytic subunit 3 (proteasome beta 5 subunit) in the immunoproteasome. |
| Proteasome subunit, beta type, 9 (large multifunctional peptidase 2) | PSMB9 | A member of the proteasome B-type family. Expression of PSMB8 is induced by gamma interferon. Replaces catalytic subunit 1 (proteasome beta 6 subunit) in the immunoproteasome. |
| Proteasome activator subunit 1 (PA28 alpha) | PSME1 | Regulator part of the immunoproteasome that processes class I MHC peptides. PSME1 gene encodes the alpha subunit of the 11S regulator, one of the two 11S subunits that is induced by gamma-interferon. |
| Proteasome activator subunit 2 (PA28 beta) | PSME2 | Regulator part of the immunoproteasome that processes class I MHC peptides. PSME2 gene encodes the beta subunit of the 11S regulator, one of the two 11S subunits that is induced by gamma-interferon. |
| Negative regulator of ubiquitin-like proteins 1 | NUB1 | Facilitator of proteasome degradation. Interacts with and negatively regulates NEDD8, a ubiquitin-like protein that covalently conjugates to cullin family members. |
| Leucine aminopeptidase 3 | LAP3 | Trims epitope precursors from the amino terminus to their correct size for MHC class I binding to enhance antigen presentation. |
| Beta-2-microglobulin | B2M | Beta chain of MHC class I molecules |
| **APOPTOSIS RELATED** | | |
| B-cell CLL/lymphoma 2 | BCL2 | Encodes an integral outer mitochondrial membrane protein that blocks the apoptotic death of some cells such as lymphocytes. |
| B-cell translocation gene 1, anti-proliferative | BTG1 | Bcl-2-regulated mediator of apoptosis. Its induction contributes to antisense Bcl-2-mediated cytotoxic effects. |
| BCL2-associated athanogene | BAG1 | Enhances the anti-apoptotic effects of BCL2 |
| Fas/CD95 | FAS | Fas introduces apoptosis-inducing signals into cells and is implicated in peripheral lymphocyte regulation, elimination of autoreactive cells and virus-infected cells, Activates NF-kB, MAPK3/ERK1, and MAPK8/JNK. Plasma levels may be a marker for chronic hepatitis C infections. |
| Inhibitor of kappa light polypeptide gene enhancer in B-cells, kinase β/ IKK2/ IKK- β | IKBKB | Phosphorylates multiple p65 sites, as well as in an IkB-p65 complex. Slightly reduces TNF-α and IL-1 β-induced NF-kB activation. Is involved in NF-kB mediated gene transcription of pro-inflammatory cytokines. HCV core suppresses IKK signalsome activity through binding to IKK-β, which blunts COX-2 expression in macrophages. |
| Inhibitor of kappa light polypeptide gene enhancer in B-cells, kinase epsilon | IKBKE | IKBKE is a noncanonical I-kappa-B kinase (IKK) that is essential for regulating antiviral signaling pathways. |
| Nuclear factor of kappa light polypeptide gene enhancer in B-cells 2 (p49/p100) | NFKB2 | Encodes one of the NF-kappaB family of transcription factors that have key roles in regulating immunity, stress responses, apoptosis, and differentiation. |
| Inhibitor of kappa light polypeptide gene enhancer in B-cells, kinase gamma | IKBKG | This gene encodes the regulatory subunit of the inhibitor of kappaB kinase (IKK) complex, which activates NF-kappaB resulting in activation of genes involved in inflammation, immunity, cell survival, and other pathways |
| TRAF family member-associated NFKB activator/ I-TRAF | TANK | Transduces signals from members of the tumor necrosis factor receptor superfamily. Can bind to TRAF1, TRAF2, or TRAF3, thereby inhibiting TRAF function by sequestering the TRAFs in a latent state in the cytoplasm. TANK may be a critical adaptor that regulates the assembly of the TANK-binding kinase 1-inducible IkB kinase complex with upstream signaling molecules in multiple antiviral pathways. |
| TNF receptor-associated factor 6 | TRAF6 | Mediates signals from CD40, TNFSF11/RANCE and IL-1 Interacts with IRAK1/IRAK, SRC and PKCzeta, which provides a link between distinct signaling pathways. Signal transducer in the NF-kB pathway that activates IkB kinase (IKK) in response to proinflammatory cytokines through interaction with ubiquitin conjugating enzymes. |
| CASP8 and FADD-like apoptosis regulator | CFLAR | Is structurally similar to caspase-8. However, the encoded protein lacks caspase activity and appears to be itself cleaved into two peptides by caspase-8. |
| TNFRSF1A-associated via death domain | TRADD | Encodes a death domain containing adaptor molecule that interacts with TNFRSF1A/TNFR1 and mediates programmed cell death signaling and NF-kappaB activation. This protein binds adaptor protein TRAF2, reduces the recruitment of inhibitor-of-apoptosis proteins (IAPs) by TRAF2, and thus suppresses TRAF2 mediated apoptosis. This protein can also interact with receptor TNFRSF6/FAS and adaptor protein FADD/MORT1, and is involved in the Fas-induced cell death pathway. |
| **CYTOSKELETON AND EXTRACELLULAR MATRIX RELATED** | | |
| Intercellular adhesion molecule 1 (CD54) | ICAM1 | Is typically expressed on endothelial cells and cells of the immune system. ICAM1 binds to integrins of type CD11a / CD18, or CD11b / CD18. Serum levels of sICAM-1 are significantly higher in HCV-RNA positive patients |
| Selectin L (lymphocyte adhesion molecule 1) | SELL | Cell surface component. A member of a family of adhesion/homing receptors which play important roles in leukocyte-endothelial cell interactions. |
| Catenin (cadherin-associated protein), beta 1, 88kDa | CTNNB1 | Adherens junction protein. Coactivator with Lef/Tcf transcription. Wnt signal increases the cytoplasmic free pool of beta-catenin that translocates into the nucleus. Is also activated by integrin-linked kinase (ILK) pathway. |
| Lectin, galactoside-binding, soluble, 9 (galectin 9) | LGALS9 | S-type lectin implicated in modulating cell-cell and cell-matrix interactions. |
| Syndecan binding protein (syntenin) | SDCBP | Affects cytoskeletal-membrane organization, cell adhesion, protein trafficking, and the activation of transcription factors. |
| Thymosin beta 4, X-linked | TMSB4X | An actin sequestering protein which plays a role in regulation of actin polymerization. The protein is also involved in cell proliferation, migration, and differentiation. |
| **PROTEASES AND RELATED PROTEINS** | | |
| Matrix metallopeptidase 9 (gelatinase B) | MMP9 | Degrades type IV and V collagens. Is involved in IL-8-induced mobilization of hematopoietic progenitor cells from bone marrow. MMP-9 gene polymorphisms account for some of the variability in the progression of HCV-related chronic liver diseases. |
| ADAM metallopeptidase domain 9 (meltrin gamma) | ADAM9 | Modulator of cell-cell and cell-matrix interactions. Involved in monocyte fusion. |
| Plasminogen activator, urokinase receptor | PLAUR | Receptor for urokinase plasminogen activator. Influences many processes related to localized degradation of the extracellular matrix. |
| Plasminogen activator inhibitor-1 (PAI-1) | SERPINE1 | Involved in wound healing, atherosclerosis, obesity and insulin resistance, tumor angiogenesis, chronic stress and fibrosis. It is not always clear if these functions depend on the antiproteolytic activity of PAI-1, on its binding to vitronectin or on its intereference with cellular migration or matrix binding. |
| **CYTOPLASMIC ENZYMES** | | |
| Guanosine monophosphate reductase | GMPR | Catalyzes the irreversible and NADPH-dependent reductive deamination of GMP to IMP. Plays a critical role in re-utilization of free intracellular bases and purine nucleosides. |
| Tryptophanyl-tRNA synthetase | WARS | Aminoacyl-tRNA synthetases catalyze the aminoacylation of tRNA by their cognate amino acid. |
| Tyrosyl-tRNA synthetase | YARS | Aminoacyl-tRNA synthetases catalyze the aminoacylation of tRNA by their cognate amino acid. Cytokine activities have also been observed for the human tyrosyl-tRNA synthetase, as its N-terminal fragment is an interleukin-8-like cytokine, whereas the released C-terminal fragment is an EMAP II-like cytokine. |
| Threonyl-tRNA synthetase | TARS | Aminoacyl-tRNA synthetases catalyze the aminoacylation of tRNA by their cognate amino acid. |
| Glutathione peroxidase 1 | GPX1 | Detoxifies hydrogen peroxide |
| Lipase A, lysosomal acid, cholesterol esterase | LIPA | Functions in the lysosome to catalyze the hydrolysis of cholesteryl esters and triglycerides. |
| Cytochrome c oxidase assembly homolog | COX17 | This nuclear gene encodes a protein which is not a structural subunit, but may be involved in the recruitment of copper to mitochondria for incorporation into the COX apoenzyme. |
| **MISCELLANEOUS** | | |
| GTP binding protein 2 | GTPBP2 | GTP-binding protein |
| Ras homolog gene family, member C | RHOC | A member of the Rho family of small GTPases |
| Adaptor-related protein complex 3, mu 2 subunit | AP3M2 | A subunit of the heterotetrameric adaptor-related protein complex 3 (AP-3) that plays a role in protein trafficking to lysosomes and specialized organelles. |
| SP100 nuclear antigen | SP100 | This gene encodes a subnuclear organelle and major component of the PML (promyelocytic leukemia)-SP100 nuclear bodies. PML and SP100 are covalently modified by the SUMO-1 modifier, which is considered crucial to nuclear body interactions. The encoded protein binds heterochromatin proteins. Genes that are upregulated by SP100 have antimigratory or antiangiogenic properties. |
| Single-stranded DNA binding protein 1 | SSBP1 | is a housekeeping gene involved in mitochondrial biogenesis. It is also a subunit of a single-stranded DNA (ssDNA)-binding complex involved in the maintenance of genome stability. |
| DEAH (Asp-Glu-Ala-His) box polypeptide 9 (DHX9) | DHX9 | DEAD box protein with RNA helicase activity. It may participate in melting of DNA:RNA hybrids, such as those that occur during transcription, and may play a role in X-linked gene expression. Interacts with NF-kappa B p65 and functions as a transcriptional coactivator. |
| Transporter 1, ATP-binding cassette, sub-family B (MDR/TAP) | TAP1 | Involved in the pumping of degraded cytosolic peptides across the endoplasmic reticulum into the membrane-bound compartment where class I molecules assemble |
| Transporter 2, ATP-binding cassette, sub-family B (MDR/TAP) [ | TAP2 | Involved in antigen presentation. |
| ATPase, H+ transporting, lysosomal 21kDa, V0 subunit b | ATP6V0B | A component of vacuolar ATPase (V-ATPase), a multisubunit enzyme that mediates acidification of eukaryotic intracellular organelles. |
| Split hand/foot malformation (ectrodactyly) type 1 | SHFM1 | Directly interacts with BRCA2. May play a role in the completion of the cell cycle. |
| CD58 molecule (LFA-3) | CD58 | Plays a central role for naive and memory T helper cells during the early phase of an immune response. The LFA-3/CD2 pathway initiates strong antigen-independent cell adhesion, substantial expansion of naive T helper cells, and induction of large amounts of IFN-gamma in memory cells. The level of CD58 molecule (in both serum and PBMC form) of patients with hepatitis B is related to the degree of liver damage |
| CD47 molecule | CD47 | Membrane protein involved in the increase in intracellular calcium concentration that occurs upon cell adhesion to extracellular matrix. A receptor for the C-terminal cell binding domain of thrombospondin. May play a role in membrane transport and signal transduction. |
| CD81 molecule | CD81 | A cell surface glycoprotein that is known to complex with integrins. This protein appears to promote muscle cell fusion and support myotube maintenance. Also it may be involved in signal transduction. |
| Myeloid cell nuclear differentiation antigen | MNDA | Is detected only in nuclei of cells of the granulocyte-monocyte lineage. Is significantly upregulated in human monocytes exposed to interferon alpha. |
| Cytochrome c oxidase assembly homolog | COX17 | The terminal component of the mitochondrial respiratory chain that catalyzes the electron transfer from reduced cytochrome c to oxygen. |
| Tripartite motif containing 14 | TRIM14 | A member of the tripartite motif (TRIM) family. Function is unclear |
| Tripartite motif containing 22 | TRIM22 | This protein localizes to the cytoplasm and its expression is induced by interferon. The protein down-regulates transcription from the HIV-1 LTR promoter region, suggesting that function of this protein may be to mediate interferon's antiviral effects. |
| Tripartite motif containing 26 | TRIM26 | A member of the tripartite motif (TRIM) family. Function is unclear |
| Tripartite motif containing 34 | TRIM34 | A member of the tripartite motif (TRIM) family. Function is unclear. Expression of this gene is up-regulated by interferon. |
